# pJRES Binning Algorithm (JBA): a new method to facilitate the recovery of metabolic information from pJRES ^1^H NMR spectra

**DOI:** 10.1093/bioinformatics/bty837

**Published:** 2018-10-23

**Authors:** Andrea Rodriguez-Martinez, Rafael Ayala, Joram M Posma, Nikita Harvey, Beatriz Jiménez, Kazuhiro Sonomura, Taka-Aki Sato, Fumihiko Matsuda, Pierre Zalloua, Dominique Gauguier, Jeremy K Nicholson, Marc-Emmanuel Dumas

**Affiliations:** 1 Division of Integrative Systems Medicine and Digestive Diseases, Department of Surgery and Cancer, Imperial College London, London, UK; 2 Department of Epidemiology and Biostatistics School of Public Health, Imperial College London, London, UK; 3 Section of Structural Biology, Department of Medicine, Shimadzu Corporation, Kyoto, Japan; 4 Life Science Research Center, Technology Research Laboratory, Shimadzu Corporation, Kyoto, Japan; 5 Center for Genomic Medicine, Kyoto University Graduate School of Medicine, Kyoto, Japan; 6 School of Medicine, Lebanese American University, Beirut, Lebanon; 7 Cordeliers Research Centre, INSERM UMR_S, Paris, France

## Abstract

**Motivation:**

Data processing is a key bottleneck for ^1^H NMR-based metabolic profiling of complex biological mixtures, such as biofluids. These spectra typically contain several thousands of signals, corresponding to possibly few hundreds of metabolites. A number of binning-based methods have been proposed to reduce the dimensionality of 1 D ^1^H NMR datasets, including statistical recoupling of variables (SRV). Here, we introduce a new binning method, named JBA (“pJRES Binning Algorithm”), which aims to extend the applicability of SRV to pJRES spectra.

**Results:**

The performance of JBA is comprehensively evaluated using 617 plasma ^1^H NMR spectra from the FGENTCARD cohort. The results presented here show that JBA exhibits higher sensitivity than SRV to detect peaks from low-abundance metabolites. In addition, JBA allows a more efficient removal of spectral variables corresponding to pure electronic noise, and this has a positive impact on multivariate model building

**Availability and implementation:**

The algorithm is implemented using the MWASTools R/Bioconductor package.

**Supplementary information:**

Supplementary data are available at *Bioinformatics* online.

## 1 Introduction

Proton nuclear magnetic resonance (^1^H NMR) spectroscopy is one of the analytical techniques of choice for metabolic phenotyping. Benefiting from very high reproducibility, high quantitative accuracy and minimal sample preparation ^1^H NMR spectroscopy has been successfully applied in various fields including, molecular epidemiology, toxicology and drug discovery (Elliott *et al.*, 2015; Nicholson *et al.*, 2002).

To date, most ^1^H NMR-based metabotyping studies have relied on one-dimensional (1D) experiments, as they require relatively short acquisition time and therefore maximize the throughput. However, a major limitation of 1 D NMR spectroscopy is the considerable overlap of spectral resonances, which reduces the number of metabolites that can be reliably identified and quantified (Nicholson *et al.*, 1995). J-resolved (JRES) spectroscopy efficiently alleviates the problem of spectral congestion by spreading the overlapped resonances into a second dimension (Aue *et al.*, 1976). The projection of a JRES spectrum along the chemical shift axis yields a virtual broadband decoupled spectrum (pJRES), which can be treated as a typical 1 D spectrum for subsequent statistical analyses (Rodriguez-Martinez *et al.*, 2017a).

^1^H NMR spectroscopy of biofluids (e.g. plasma or urine) leads to complex spectra composed of thousands of variables, corresponding to probably few hundreds of metabolites, amongst which less than one hundred can be typically assigned in a single NMR spectrum (Nicholson *et al.*, 1995). The high dimensionality inherent to ^1^H NMR data makes it challenging to extract meaningful biological information, and leads to a high burden of multiple-testing when performing univariate statistical tests. In order to reduce data dimensionality, binning (also known as bucketing) is commonly used (Holmes *et al.*, 1994; Spraul *et al.*, 1994). In binning, the spectra are divided into spaced chemical shift regions (i.e. “bins”) and the area under each bin is used, instead of the individual intensities. Although computationally simple and fast, this approach tends to lack accuracy, particularly in crowded spectral regions where overlapped peaks are likely to fall within the same bin.

A number of computational algorithms have been proposed to overcome this drawback, such as Gaussian binning (Anderson *et al.*, 2008), adaptive binning (Davis *et al.*, 2007) and adaptive intelligent binning (De Meyer *et al.*, 2008). Although these methods clearly outperform standard binning, they are computationally demanding (i.e. unsuited for datasets with a large number of samples) and/or are not implemented in open-source software programs. Another alternative method is statistical recoupling of variables (SRV) (Blaise *et al.*, 2009). SRV takes advantage of the collinearity of NMR variables across a set of spectra (Cloarec *et al.*, 2005) to form clusters (i.e. bins) of adjacent variables following the direction of the highest covariance to correlation ratio. Since both covariance and correlation can be easily computed, SRV is a fast method. This “clever-binning” algorithm has been proved to be a valuable tool in numerous 1 D ^1^H NMR based metabotyping studies (Cazier *et al.*, 2012; Dao *et al.*, 2016; Dumas *et al.*, 2017; Gu *et al.*, 2016).

Here, we propose a new binning method, named JBA (“pJRES Binning Algorithm”), which aims to extend the applicability of the SRV algorithm to the 1 D projections of JRES spectra. We evaluate the performance of the JBA algorithm in comparison with the use of SRV and standard binning (SB). On the basis of the assessment of several objective criteria, the results presented here demonstrate that, compared to SRV and SB, JBA exhibits: (i) increased selectivity to discriminate between metabolic signals and electronic noise; (ii) enhanced sensitivity to detect peaks from low-abundance metabolites that typically overlap with the tails of high intensity pJRES peaks.

## 2 Materials and methods

### 2.1 Metabonomic data

We used a subset of plasma samples from the FGENTCARD cohort profiled by ^1^H NMR spectroscopy (*n* = 617) and by gas-chromatography coupled to mass spectrometry (GC-MS, *n* = 35) as described in (Rodriguez-Martinez *et al.*, 2017a)

Briefly, plasma samples (*n* = 617) were analyzed using a Bruker Avance III 600 MHz spectrometer (Bruker Biospin Ltd, Germany) operating at 310 K. The pulse sequence used to acquire the JRES spectra takes the form: -RD-90°-*t*_1_-180°-*t*_1_-acquire FID, where RD is the relaxation delay and *t*_1_ is the increment delay. JRES spectra were acquired using 4 scans per increment over 40 increments, which were collected in 8000 data points using spectral windows of 16.6 ppm in F2 and 78 Hz in F1. Following spectral acquisition, the data were automatically processed using TopSpin 3.2 with Icon (Bruker Biospin Ltd, Germany). Zero-filling by a factor of 2 was included in F2 and the digital resolution was increased to 256 in F1 by zero-filling. Apodization of JRES spectra using a sine-bell function was applied in both F1 and F2 dimensions prior to Fourier transformation. The JRES spectra were then tilted, symmetrized and skyline projected to obtain the pJRES spectra. The spectra were calibrated to the α-glucose anomeric signal at *δ* 5.23 (Pearce *et al.*, 2008) and spectral regions exhibiting considerable peak shifts were manually aligned (Veselkov *et al.*, 2009). Metabolite assignment was performed using an in-house database (Nicholson *et al.*, 1995), correlation-based analysis (Cloarec *et al.*, 2005; Crockford *et al.*, 2006; Posma *et al.*, 2012), a range of 2 D NMR experiments and spiking experiments with authentic commercial standards. The raw ^1^H NMR files are available from MetaboLights (Haug *et al.*, 2013) under accession number MTBLS540. The pre-processed ^1^H NMR spectra are available from (https://github.com/AndreaRMICL/NMR_Metabonomics_data).

A subset of plasma samples (*n* = 35) was also profiled by GC-MS using a GCMS-QP2010 spectrometer (Shimadzu, Kyoto, Japan), as previously described (Rodriguez-Martinez *et al.*, 2017a). GC-MS data processing was carried out using GCMSsolution 2.71 (Shimadzu, Kyoto, Japan). Assignment of chromatographic peaks was performed using the NIST library or Shimadzu GC/MS database, and further confirmed with authentic commercial standards.

### 2.2 JBA algorithm

#### 2.2.1 Parameters

**Fig. 1.**
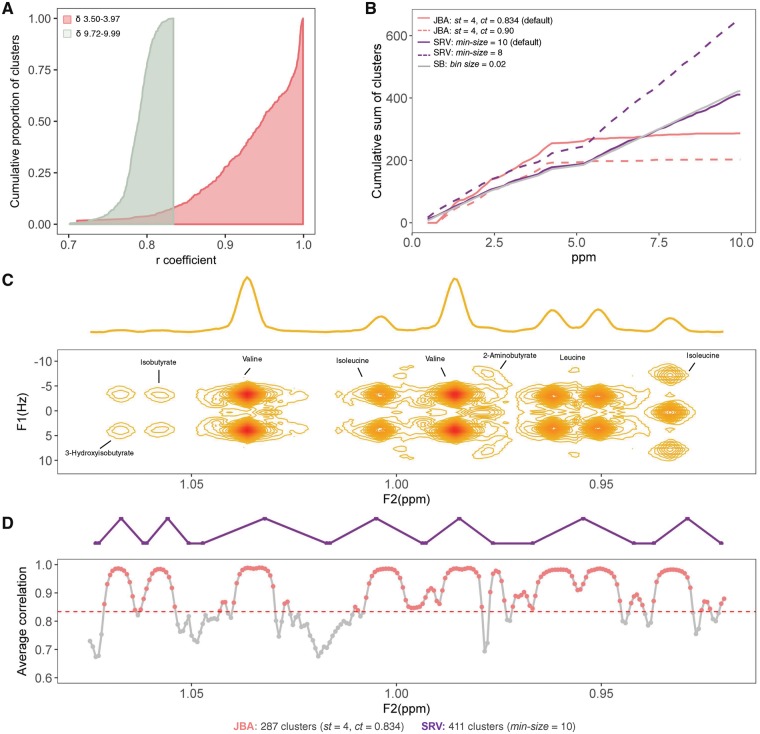
Overview of the JBA algorithm using pJRES spectra of plasma samples from the FGENTCARD cohort (*n* = 617). (**A**) Comparison of correlations between *st* (*st* = 4) adjacent variables in a spectral region dominated by metabolic signals (*δ* 3.50–3.97, coral) and in a noise region (*δ* 9.72–9.99, green). (**B**) Cumulative sum of clusters detected along the chemical shift axis in JBA, SRV and SB spectra. (**C**) 2D JRES ^1^H NMR spectrum of a pooled sample displayed as a contour plot underneath the corresponding skyline 1D projection. (**D**) Pseudo-NMR spectrum showing the correlation between *st* (*st* = 4) adjacent NMR variables along the chemical shift axis, where clusters with correlation above *ct* (*ct* = 0.834) are represented in coral. The purple line represents the SRV clusters formed in this spectral region

The JBA algorithm is implemented using the MWASTools R/Bioconductor package (Rodriguez-Martinez *et al.*, 2018). There are four main user-defined parameters: ***st*, *ct*, *cm*, *int*.** The ***st*** value establishes the minimum cluster size (i.e. minimum number of NMR variables that define a cluster). This parameter depends on the resolution (i.e. number of data points covering the peak width) of the raw NMR data. The ***ct*** value indicates the minimum average correlation between the ***st*** variables of a given cluster to be considered a metabolic cluster. This parameter allows discrimination between NMR signals corresponding to metabolic resonances and NMR signals corresponding to electronic noise. The value given to ***ct*** can be established by comparing the correlations of ***st*** adjacent variables in a spectral region dominated by metabolic resonances (e.g*. δ* 3.50–3.97) and in a spectral region dominated by electronic noise (e.g. *δ* 9.72–9.99), and selecting the correlation coefficient where the cumulative proportion of noise clusters is cdf =1 (Fig. 1A). The value of ***cm*** indicates the correlation method (i.e. Spearman or Pearson) and ***int*** indicates whether the intensity of a given cluster is calculated as the sum, maximum, mean, or median of all the intensities within that cluster.

#### 2.2.2 Steps

First, the algorithm scans the NMR spectra (from low to high frequencies) and calculates the average correlation of ***st*** adjacent variables, using a sliding window of size one. This means that a given cluster *i* starts at the NMR variable *i* and finishes at NMR variable with *i* + (*st* − 1).

Second, the vector of average correlations can be represented as a pseudo-NMR spectrum, displaying the average correlation values in the *y*-axis and the chemical shifts in the *x*-axis (Fig. 1D). This correlation-based spectrum is then scanned to identify local maxima passing the ***ct*** threshold. Each of these local maxima is considered as the optimal correlation-based cluster of size ***st*** of the corresponding NMR peak. These optimal clusters are used as seeds that are expanded by progressively aggregating upfield and downfield NMR variables, as long as the following criteria are met: (i) the average correlation of the cluster remains equal or above ***ct***; and (ii) for a given upfield variable (*v_i_*), correlation (*v_i_*, *v_i_*_+1_) needs to be equal or higher than correlation (*v_i_*, *v_i_*_-1_); or (iii) for a given downfield variable (*v_i_*), correlation (*v_i_*, *v_i_*_+1_) needs to be equal or lower than correlation (*v_i_*, *v_i_*_-1_).

Finally, the intensity of each cluster is calculated as the sum, median, mean or maximum intensity of all variables within the cluster. Notice that due to misalignments/signal overlap, it is possible that a single peak is split into several clusters. These clusters can be detected based on a given correlation threshold and integrated as a single cluster.

### 2.3 SRV algorithm

The SRV algorithm was implemented using the mQTL.NMR R/Bioconductor package (Hedjazi *et al.*, 2015). The main user-defined parameter in SRV is minsize**,** which establishes the minimum number of variables that define a metabolic cluster. This parameter represents the number of variables required to sample a well-defined singlet in an NMR spectrum, which depends on the resolution of the raw spectra.

Briefly, the SRV algorithm involves calculating the spectral dependency landscape as the covariance/correlation ratio between adjacent variables along the chemical shift axis (moving from low to high frequencies). The spectral dependency landscape is then scanned to identify local minima of covariance/correlation ratio, which correspond to the cluster edges. Clusters are retained if they contain at least minsize variables; otherwise they are neglected. The intensity of each cluster can be calculated as the sum, mean, median or maximum intensity of all the variables within the cluster. Finally, neighbouring clusters with a sufficient level of correlation (Pearson correlation > 0.90) are aggregated into “superclusters”, representing NMR signals.

## 3 Results

### 3.1 Application of JBA

The current full resolution (FR) pJRES spectra were composed of 12 273 NMR variables in the spectral window *δ* 0.40–10.00 (excluding water and EDTA signals). These NMR variables are likely to correspond to less than 100 assignable plasma metabolites, that is, two orders of magnitude less than the input variables. JBA was applied to reduce the dimensionality of pJRES spectra and facilitate the recovery of relevant metabolic information.

The JBA parameters were set to enable the detection of metabolic clusters composed of at least four adjacent NMR variables (***st*** = 4), with average correlation equal or above 0.834 (***ct*** = 0.834). The ***ct*** threshold is a crucial parameter to discriminate between metabolic signals and noise signals. Using a too lenient ***ct*** value might lead to the inclusion of clusters corresponding to pure electronic noise; while a too stringent ***ct*** value might result in loss of metabolic information. Here, the ***ct*** value was established by comparing the degree of collinearity of adjacent NMR variables in a spectral region enriched by metabolic signals (*δ* 3.50–3.97) and in a spectral region dominated by electronic noise (e.g. *δ* 9.72–9.99) (Fig. 1A, Supplementary Fig. S1). The intensity of each cluster was calculated as the sum of the intensities of all the variables within the cluster. Neighbouring clusters with correlation above 0.90 were integrated into a single cluster. In total, 287 JBA clusters were detected, mostly in the spectral window *δ* 0.82–5.30, where the vast majority of endogenous plasma metabolites resonate (Nicholson *et al.*, 1995) (Fig. 1B). A few clusters were also detected in higher frequency regions, including those corresponding to tyrosine (*δ* 6.88, 7.18) and formate (*δ* 8.45). SRV applied to the same pJRES dataset, using the default cluster size (i.e. minsize) of 10 variables, resulted in 411 clusters. Similarly to SB spectra, more than half of SRV clusters were detected in noise regions, with the proportion of noise-based clusters increasing with lower minsize values (e.g. minsize = 8) (Fig. 1B). This is due to the fact that SRV does not take into account that a certain degree of collinearity also exists in noise “peaks”, especially in pJRES spectra where the noise is not truly random (Fig. 1A, Supplementary Fig. S3).

The principle behind the JBA approach is exemplified in Figure 1C and D. Each of the local maxima in the correlation-based spectrum is considered as the most representative cluster of the corresponding NMR peak, which can be further expanded by aggregating highly correlated neighbouring NMR variables (Supplementary Fig. S2). While SRV focuses on high intensity NMR peaks, JBA also detects low intensity metabolic signals, which might be of considerable clinical interest (e.g. 2-aminobutyrate).

### 3.2 Assignment of JBA spectra

Many of the ^1^H NMR peaks detected in biofluid spectra are unknown, and metabolite assignment is a complex and time-consuming task (Dona *et al.*, 2016; Nagana Gowda *et al.*, 2015; Posma *et al.*, 2017). We previously introduced a strategy for semi-automated annotation of ^1^H NMR peaks, based on cross-correlations with GC-MS metabolites (Rodriguez-Martinez *et al.*, 2017a). By using this strategy, in combination with other statistical tools (Cloarec *et al.*, 2005; Posma *et al.*, 2012) and additional 2 D NMR experiments, 31 metabolites were identified in JBA spectra (Supplementary Table S1). These metabolites are involved in a wide range of biochemical pathways, from carbohydrate, amino acid and lipid metabolism; and some of them are products of microbial metabolism (Rodriguez-Martinez *et al.*, 2017b). Since the composition of plasma is under homeostatic control and metabolite concentrations are relatively stable, the annotated JBA clusters are expected to be reproducible across datasets.

### 3.3 Evaluation of JBA performance via cross-correlations with GC-MS metabolites

**Fig. 2.**
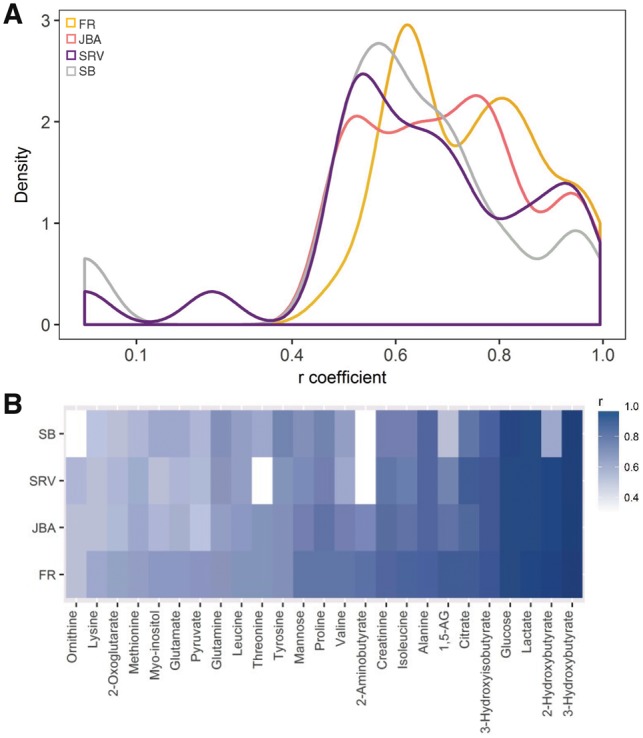
Evaluation of specificity of JBA clusters *via* cross-correlations with GC-MS metabolites (*n* = 35). (**A**) Kernel density curves of coefficients of correlation between 25 GC-MS metabolites and matched NMR signals in FR (yellow), JBA (coral), SRV (purple) and SB (grey) spectra. (**B**) Heat-map showing the coefficients of correlation between 25 GC-MS metabolites and matched NMR signals in FR, JBA, SRV and SB spectra. Abbreviations: ND indicates not detected

Next, the performance of the JBA algorithm was assessed through cross-correlations (Crockford *et al.*, 2006) with GC-MS metabolites. Spearman correlations were computed between ^1^H NMR variables and 25 GC-MS metabolites, whose identities in the ^1^H NMR spectrum were confirmed by additional 2 D and spike-in experiments. The correlations in JBA spectra were significantly higher than in SRV spectra (Wilcoxon-signed rank *P*-value = 2.19 × 10^−2^) (Fig. 2A), with remarkable differences for a number of metabolites, including 2-aminobutyrate (undetected in SRV) and threonine (Fig. 2B). These results further demonstrate the enhanced metabolic specificity and sensitivity of the JBA approach, compared to SRV. However, it should be noted that few metabolites (e.g. glutamate) exhibited considerably higher correlations in the full resolution spectra than after applying JBA (Fig. 2B), reflecting the difficulty of collapsing NMR variables in extensively overlapped spectral regions.

### 3.4 Assessment of the recovery of metabolic information in JBA spectra via PCA

**Fig. 3.**
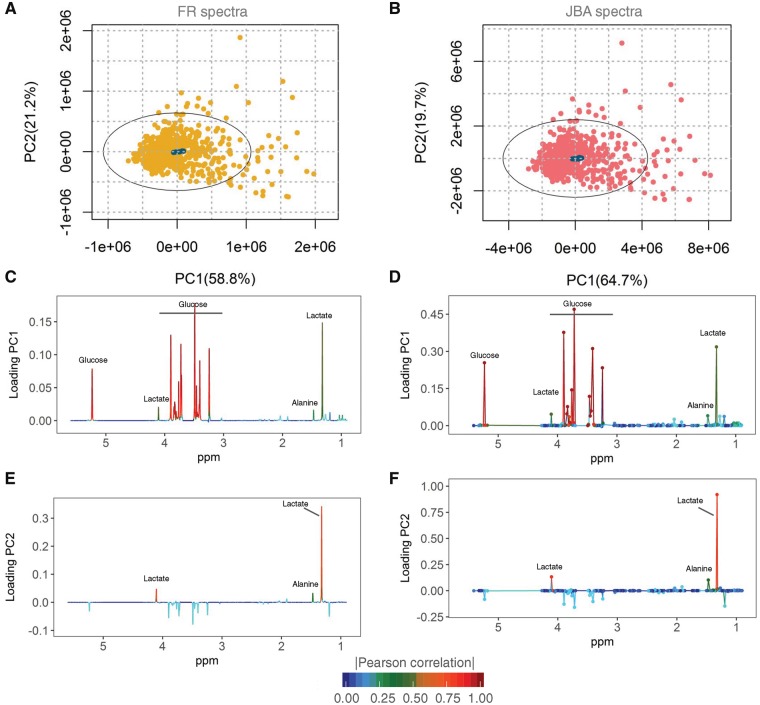
Effect of JBA pretreatment on the overall metabolic variation of the original dataset. (**A**, **B**) PCA score plots of mean-centred FR (A) and JBA (B) spectra with the QC samples (*n *=* *10) coloured in dark blue. (**C–F**) PCA loading plots corresponding to the first two principal components

The unsupervised method of principal component analysis (PCA) was used to evaluate the effect of applying JBA on the overall biological variation within the dataset. PCA models were built using mean-centred spectra from the biological samples and 10 quality control (QC) samples, prepared from a representative pool of the clinical samples and analysed regularly through the run. In the score plots from both FR and JBA models, the QC samples appeared tightly clustered in the centre of the Hotelling's ellipse (Fig. 3A and B), demonstrating that JBA does not affect the overall reproducibility of the dataset. Visual inspection of the both score plots revealed a very similar structure and common patterns. Consistently, the most discriminant metabolites identified in the loading plots from both JBA and FR models were essentially the same: glucose, lactate and alanine (Fig. 3C–F).

**Fig. 4.**
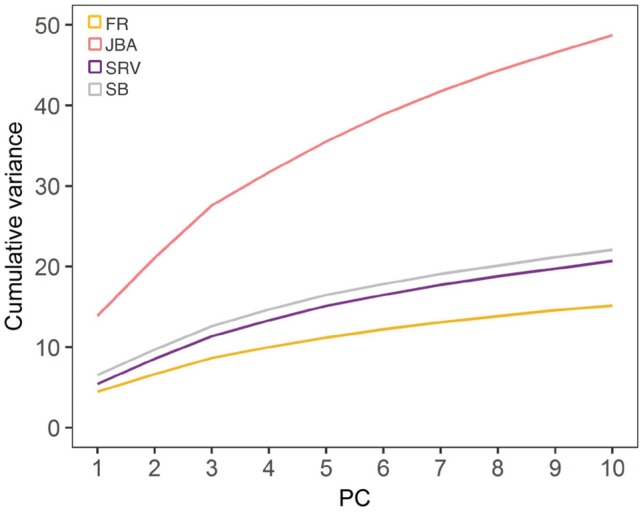
Effect of different binning methods on multivariate model building. The curves show the cumulative variance explained by the first 10 principal components using unit-variance scaled spectra

It is also noteworthy that the variance captured by the first PCs was higher in the JBA spectra than in the SRV, SB or FR datasets, particularly when using unit-variance scaled (UV) spectra (Fig. 4). This is most likely due to the fact that the JBA spectra are mostly composed by metabolic variables, eliminating the negative effect of noise variables on model building.

## 4 Conclusions

^1^H NMR spectra of biofluids are highly complex, typically consisting of tens of thousands of variables. Thus, dimensionality reduction is a critical step in ^1^H NMR data processing. Here, we introduce the binning method JBA, which aims to extend the applicability of the SRV algorithm for pJRES datasets. We showed that JBA performs adequate dimensionality reduction of pJRES spectra and outperforms both SRV and standard equidistant binning, in terms of variance explained by first PCs and cross-correlations with GC-MS data. JBA is more sensitive to detect low intensity metabolic peaks, which are often neglected or integrated with larger peaks in SRV.

Another major advantage is that in JBA spectra each cluster usually corresponds with a metabolic peak, eliminating to a large extent noise signals and their negative influence on subsequent statistical analysis. In addition, JBA spectra can be more efficiently combined with unit-variance scaling, which makes uncovering ^1^H NMR signals from low-concentration metabolites more straightforward. However, similar to other binning methods, JBA may select suboptimal bin edges in extensively misaligned or overlapped spectral regions.

JBA is computationally simple and fast (∼10 s for 1000 samples) and therefore it is suitable for implementation in large-scale datasets. Furthermore, JBA allows using different resolution parameters and correlations methods (i.e. Pearson or Spearman correlations) and therefore it is flexible and adaptable to different datasets in an objective manner. Overall, the results presented here show that JBA offers sought properties for pre-processing of large-scale pJRES datasets.

### Author contributions

A.R.-M. wrote the manuscript, developed the algorithm and performed data analysis, with input from R.A., J.M.P., and M.-E.D. A.R.-M., N.H., B.J., A.L.N., K.S., and T.-A.S. ran experiments. P.Z., D.G., J.K.N., and M.-E.D. supervised the FGENTCARD study. All authors read and approved the manuscript.

## Funding

This work was supported by: Medical Research Council Doctoral Training Centre scholarship (MR/K501281/1), Imperial College scholarship (EP/M506345/1), La Caixa studentship to A.R.M; a Rutherford Fund Fellowship at Health Data Research UK (MR/S004033/1) to J.M.P; European Commission (FGENTCARD, LSHG-CT-2006-037683 to D.G. and J.K.N. NMR experiments were run in the Clinical Phenome Centre, which is supported by the NIHR Imperial Biomedical Research Centre based at Imperial College Healthcare National Health Service (NHS) Trust and Imperial College London. The views expressed are those of the author(s) and not necessarily those of the NHS, the NIHR, or the Department of Health.

*Conflict of Interest:* none declared.

## Supplementary Material

bty837_Supplementary_Material
