# Supplementary material for: pJRES Binning Algorithm (JBA): a new method to facilitate the recovery of metabolic information from pJRES 1H NMR spectra
Source: Bioinformatics. 2018 Oct 23;35(11):1916–22. doi: 10.1093/bioinformatics/bty837 (PMC6546129; doi:10.1093/bioinformatics/bty837)

# pJRES Binning Algorithm (JBA): a new method to facilitate the recovery of metabolic information from pJRES $^1$H NMR spectra

Andrea Rodriguez-Martinez[1*], Rafael Ayala[2], Joram M. Posma[3], Nikita Harvey[1], Beatriz Jiménez[1], Kazuhiro Sonomura[4,5], Taka-Aki Sato[4,5], Fumihiko Matsuda[5], Pierre Zalloua[6], Dominique Gauguier[7], Jeremy K. Nicholson[1] and Marc-Emmanuel Dumas[1*]

[1]Division of Integrative Systems Medicine and Digestive Diseases, Department of Surgery and Cancer, Imperial College London, UK; [3]Department of Epidemiology and Biostatistics, School of Public Health, Imperial College London, UK; [2]Section of Systems Biology, Department of Medicine, Imperial College London, UK; [4]Life Science Research Center, Technology Research Laboratory, Shimadzu Corporation, Kyoto, Japan; [5]Center for Genomic Medicine, Kyoto University Graduate School of Medicine, Kyoto, Japan; [6]School of Medicine, Lebanese American University, Beirut, Lebanon; [7]Cordeliers Research Centre, INSERM UMR_S 1138, Paris, France.

## Supplementary material

In this document, we provide the following information:

- Table S1 reporting the list of metabolites identified in JBA spectra.
- Figure S1 showing the relationship between *st* and *ct* parameters.
- Figure S2 describing the steps of the JBA algorithm.
- Figure S3 showing the collinearity of adjacent spectral variables in spin-echo spectra.
- Instructions for how to implement the JBA algorithm in R.

**Table S1. Metabolites identified in JBA spectra and assignment strategy.**

| Metabolite | Chemical shifts | Assignment strategy |
|---|---|---|
| 2-Hydroxybutyrate | 0.895 (t), 3.989 (dd) | SHY-GCMS, JRES, COSY, TOCSY, spike-in |
| Isoleucine | 0.931 (t), 1.001 (d) | SHY-GCMS, JRES, COSY, TOCSY, HSQC |
| Leucine | 0.954 (dd) | SHY-GCMS, JRES, COSY, TOCSY, HSQC |
| 2-Aminobutyrate | 0.974 (t) | SHY-GCMS, COSY, TOCSY, spike-in |
| Valine | 0.980 (d), 1.030 (d) | SHY-GCMS, JRES, COSY, TOCSY, HSQC |
| Isobutyrate | 1.056 (d) | JRES, spike-in |
| 3-Hydroxyisobutyrate | 1.066 (d) | SHY-GCMS, JRES, COSY, TOCSY |
| 3-Hydroxybutyrate | 1.194 (d), 2.304 (m), 2.393 (m), 4.147 (m) | SHY-GCMS, JRES, COSY, TOCSY, HSQC |
| Threonine | 1.312 (d), 4.220 (m) | SHY-GCMS, COSY, TOCSY, HSQC |
| Lactate | 1.325 (d), 4.107 (q) | SHY-GCMS, JRES, COSY, TOCSY, HSQC |
| 2-Hydroxyisobutyrate | 1.357 (s) | JRES, spike-in |
| Lysine | 1.721 (m), 1.882 (m), 3.025 (t) | SHY-GCMS, JRES, COSY, TOCSY |
| Alanine | 1.471 (d), 3.777 (q) | SHY-GCMS, JRES, COSY, TOCSY, HSQC |
| Glutamate | 2.121 (m), 2.349 (m) | SHY-GCMS, JRES, COSY, TOCSY, HSQC |
| Methionine | 2.135 (s), 2.637 (m) | SHY-GCMS, JRES, spike-in |
| Proline | 3.335 (m), 4.121 (dd) | SHY-GCMS, JRES, COSY, TOCSY |
| Glutamine | 2.427 (m) | SHY-GCMS, JRES, COSY, TOCSY, HSQC |
| Acetone | 2.224 (s) | JRES, HSQC, spike-in |
| Pyruvate | 2.366 (s) | JRES, spike-in |
| Citrate | 2.654 (d) | SHY-GCMS, JRES, COSY, TOCSY, HSQC |
| Dimethylamine | 2.722 (s) | JRES, spike-in |
| Sarcosine | 2.741 (s) | JRES, spike-in |
| Dimethylglycine | 2.917 (s) | JRES, spike-in |
| Creatine | 3.923 (s) | JRES, COSY, TOCSY |
| Creatinine | 3.039 (d), 4.047 (s) | SHY-GCMS, JRES, COSY, TOCSY |
| Ornithine | 3.050 (t) | SHY-GCMS, JRES, spike-in |
| Glucose | 3.245 (dd), 3.390 (t), 3.461 (m), 3.493 (t), 3.720 (t), 3.733 (dd), 3.761 (m), 3.831 (m), 3.908 (dd), 5.233 (d) | SHY-GCMS, JRES, COSY, TOCSY |
| 1,5-Anhydroglucitol | 3.271 (t), 3.343 (m), 3.879 (d), 3.978 (dd) | STORM, SHY-GCMS, JRES, COSY, TOCSY |
| Mannose | 5.183 (d) | SHY-GCMS, JRES, spike-in |
| Tyrosine | 6.892 (d), 7.187 (d) | JRES, COSY, TOCSY, HSQC |
| Formate | 8.454 (s) | JRES, HSQC |

Multiplicity nomenclature: singlet (s), doublet (d), triplet (t), quartet (q), multiplet (m), doublet of doublets (dd). Abbreviations: JRES, *J*-Resolved spectroscopy; SHY, statistical heterospectroscopy; STORM, statistical optimization by reference matching; GCMS, mass spectrometry coupled to gas chromatography; COSY correlation spectroscopy; TOCSY, total correlation spectroscopy; HSQC, heteronuclear single quantum coherence.

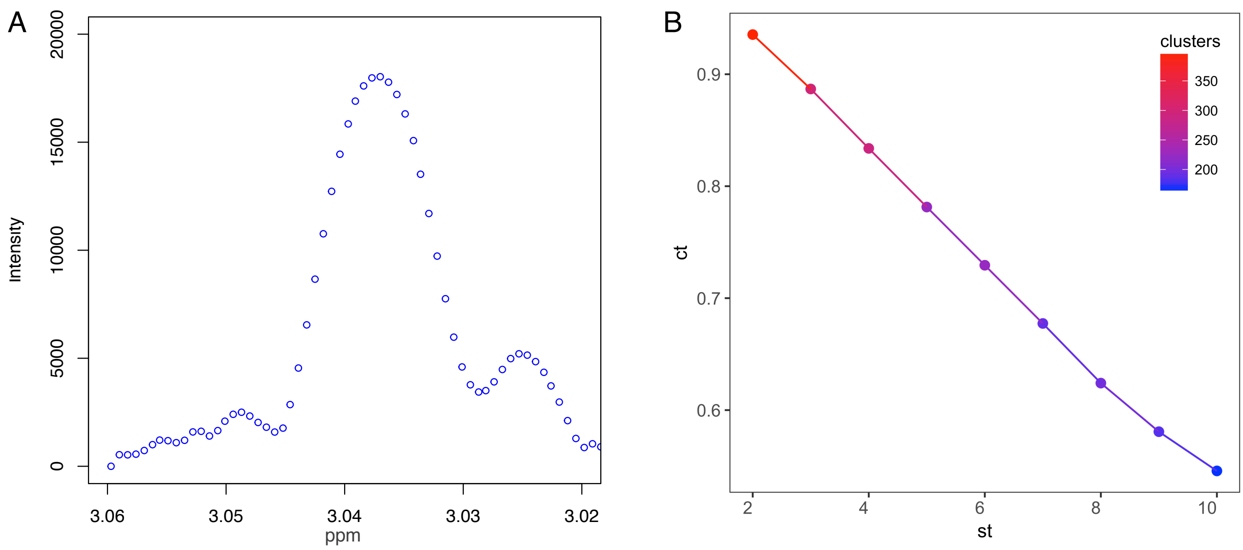


**Figure S1. Selection of *st* and *ct* parameters.** (A) The selection of the *st* value depends on the resolution of the raw spectra (i.e. number of data points covering the peak width). Selecting a supraoptimal *st* value (e.g. *st* = 10) may lead to the exclusion of low-intensity metabolic peaks close to the baseline. (B) Relationship between *st* and *ct* values, where the color indicates the number of clusters generated with each set of parameters. For each *st* value, the *ct* value was established by comparing the correlation of *st* adjacent variables in a spectral region dominated by metabolic signals with a spectral region corresponding to pure electronic noise, and selecting the correlation coefficient where the cumulative proportion of noise clusters is 1.

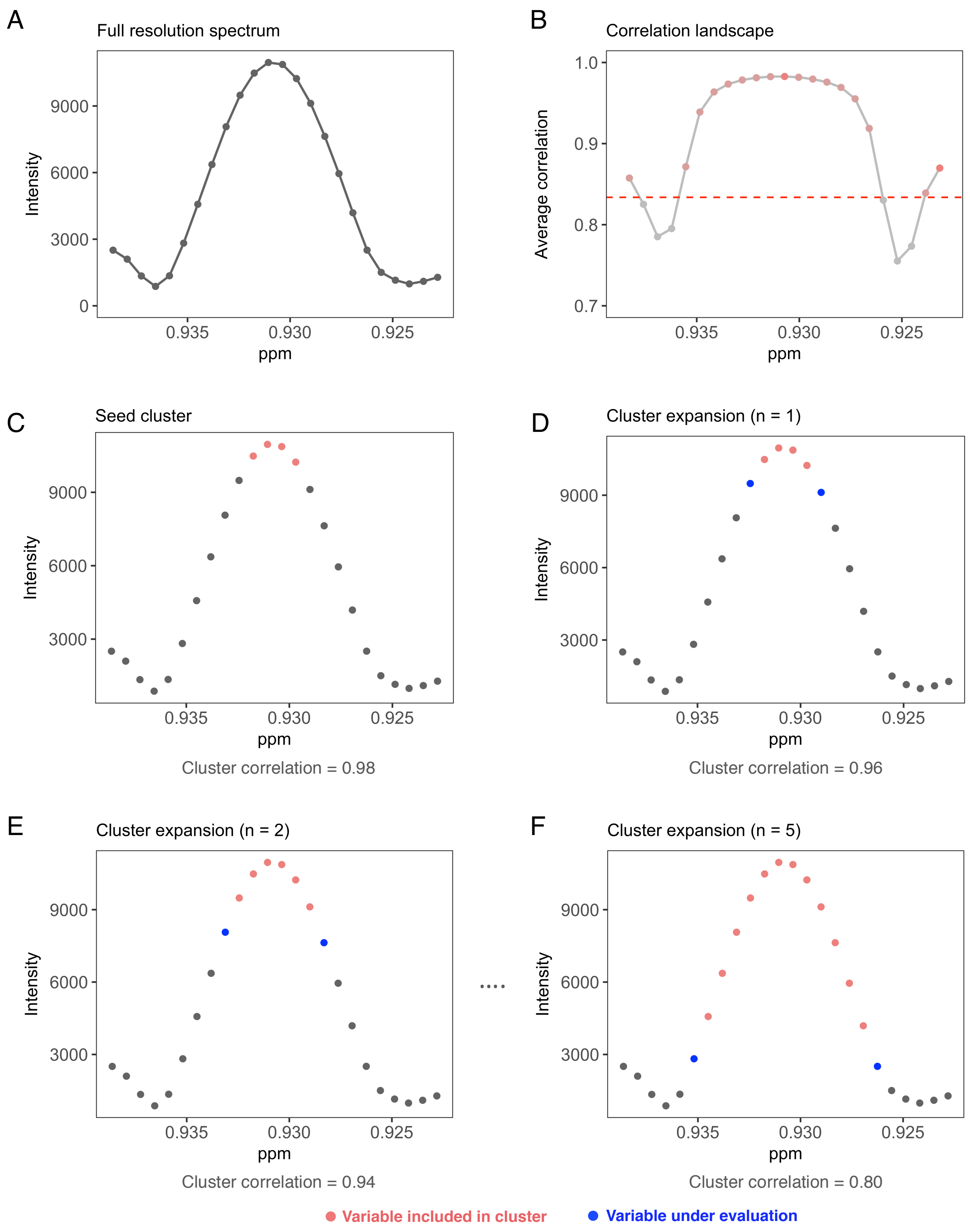


**Figure S2. Schematic representation of JBA steps.** The algorithm starts by computing the correlation of *st* (*st* = 4) adjacent variables using a sliding window of size one (B). The correlation-based landscape is then explored to identify local maxima passing the *ct* threshold (*ct* = 0.834). Each local maximum is used as a seed cluster (C), which can be further expanded by progressively aggregating upfield and downfield neighbouring variables (D-F). In this example, the cluster expansion finishes at the 5$^{th}$ iteration (F) as the cluster correlation (r = 0.80) drops below the *ct* value.

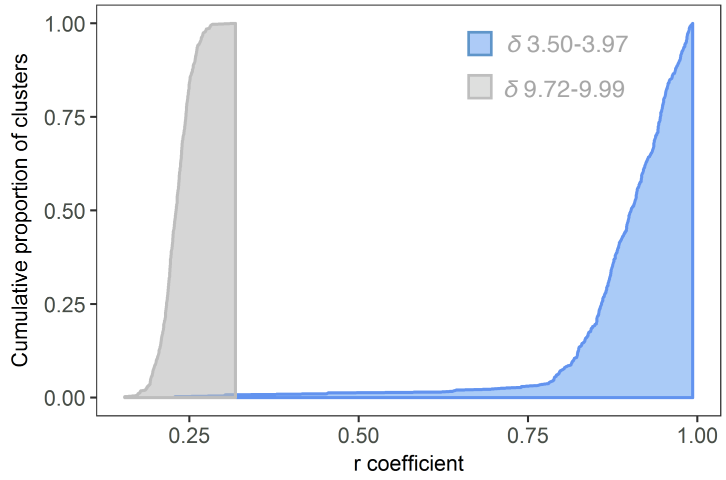


**Figure S3. Collinearity of adjacent $^{1}$H NMR variables in spin-echo spectra from 617 plasma samples from the FGENTCARD cohort.** Each point in this cumulative frequency graph corresponds to the total number of clusters whose correlation coefficient (r) is less than or equal to a given level. The grey curve corresponds to clusters generated in the spectral region δ 9.72-9.99, while the blue curve corresponds to clusters generated in the spectral region δ 3.50-3.97.

## Installation instructions

JBA binning is implemented using the MWASTools R/Bioconductor package (version > 1.5.1). For details, check: https://bioconductor.org/packages/MWASTools/

To install MWASTools, start R (or RStudio) and follow the instructions shown below:

```
# A) Install the release version of MWASTools
source("https://bioconductor.org/biocLite.R")
biocLite("MWASTools")

# B) Install the devel version of MWASTools
if (!requireNamespace("BiocManager", quietly=TRUE))
install.packages("BiocManager")
BiocManager::install("MWASTools", version = "devel")
```

# 3) Remove package

To remove the package use: remove.packages("MWASTools") and restart R.

## Get started

# Under R, and after having installed MWASTools, load the package using:

```
library(MWASTools)
```

# MWASTools contains three JBA-related functions: “JBA_binning()”, “JBA_corDistribution()”, and “JBA_plotBins()”. To get help with any of these functions (e.g. “JBA_binning()”) use the “help()” function as shown below:

```
help("JBA_binning")
```

## Implementation

To illustrate how to use the JBA-related functions from MWASTools, we use the pJRES $^{1}$H NMR dataset from (https://github.com/AndreaRMICL/NMR_Metabonomics_data). After saving the file “JBA_NMR_data.csv” in the working directory, we import it in R as shown below:

```
## Import NMR data
NMR_data <- as.matrix(read.csv("JBA_NMR_data.csv", header = TRUE, row.names = 1,
check.names = FALSE))
```

Set *ct* and *st* values

Before running the "JBA_binning()" function we need to decide the value of the two key parameters of the JBA algorithm: ***ct*** and ***st.*** Given the resolution of the raw pJRES $^{1}$H NMR spectra (see Fig. S1A), we set ***st*** (i.e. minimum bin size) to 4. In order to establish the value of ***ct***, we use the function "JBA_corDistribution()", which compares the degree of collinearity between adjacent variables in a spectral region dominated by metabolic signals (e.g. δ 3.50-3.96), and a spectral region dominated by noise (e.g. δ 9.72-9.99). Based on the output of this function, we set ***ct*** to 0.83376.

```
## Set ct value
library(ggplot2) # Useful to set limits
help(JBA_corDistribution) # Read documentation
JBA_corDistribution(NMR_data, st = 4, metabo_range = c(3.50, 3.96), noise_range =
c(9.72, 9.99)) + xlim (0.70, 1)
```

[1] "Suggested ct value: 0.83376"

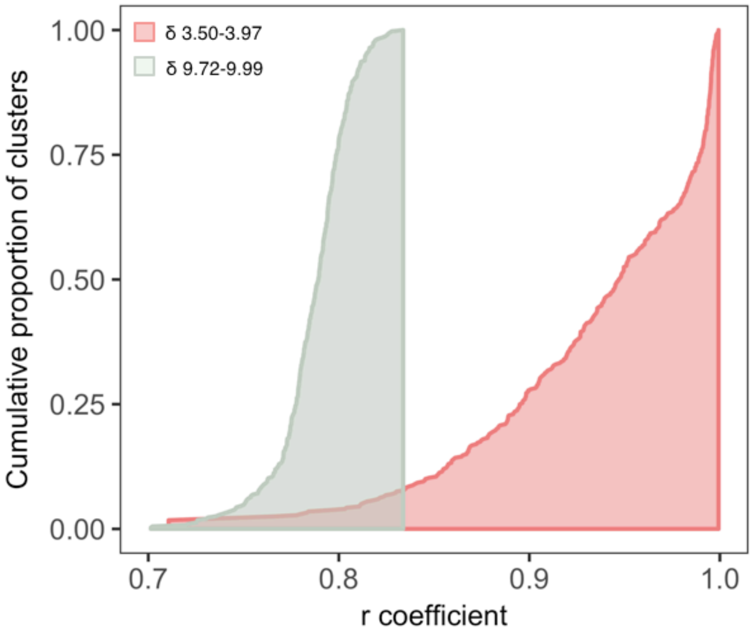


Apply JBA

Next, we apply the JBA algorithm to our pJRES spectra using the "JBA_binning()" function. As described in the reference manual, the output of "JBA_binning()" is a list containing the JBA-binned profiles, as well as information about the JBA clusters (e.g. cluster edges).

```
## Apply JBA
help(JBA_binning) # Read documentation
JBA_output <- JBA_binning(NMR_data, st = 4, ct = 0.83376)
```

```
## Get JBA spectra
JBA_spectra <- JBA_output$JBA_data
dim(JBA_spectra) # 617 samples and 287 JBA clusters
```

Visualize JBA spectra

Finally, we use the function “JBA_plotBins()” to visualize the clusters formed by the JBA algorithm. The output of this function is a plot with two panels. The upper panel shows a full-resolution pJRES spectrum with the cluster edges as vertical lines (dark-blue: start, light-blue: end). The lower panel corresponds to the correlation-based spectrum used to calculate the JBA clusters.

```
## Visualize JBA clusters
help(JBA_plotBins) # Read documentation
JBA_plotBins(NMR_JBA = JBA_output, NMR_data = NMR_data, ct = 0.83376, ref_sample = 1
00, xlim = c(0.92,0.977))
```

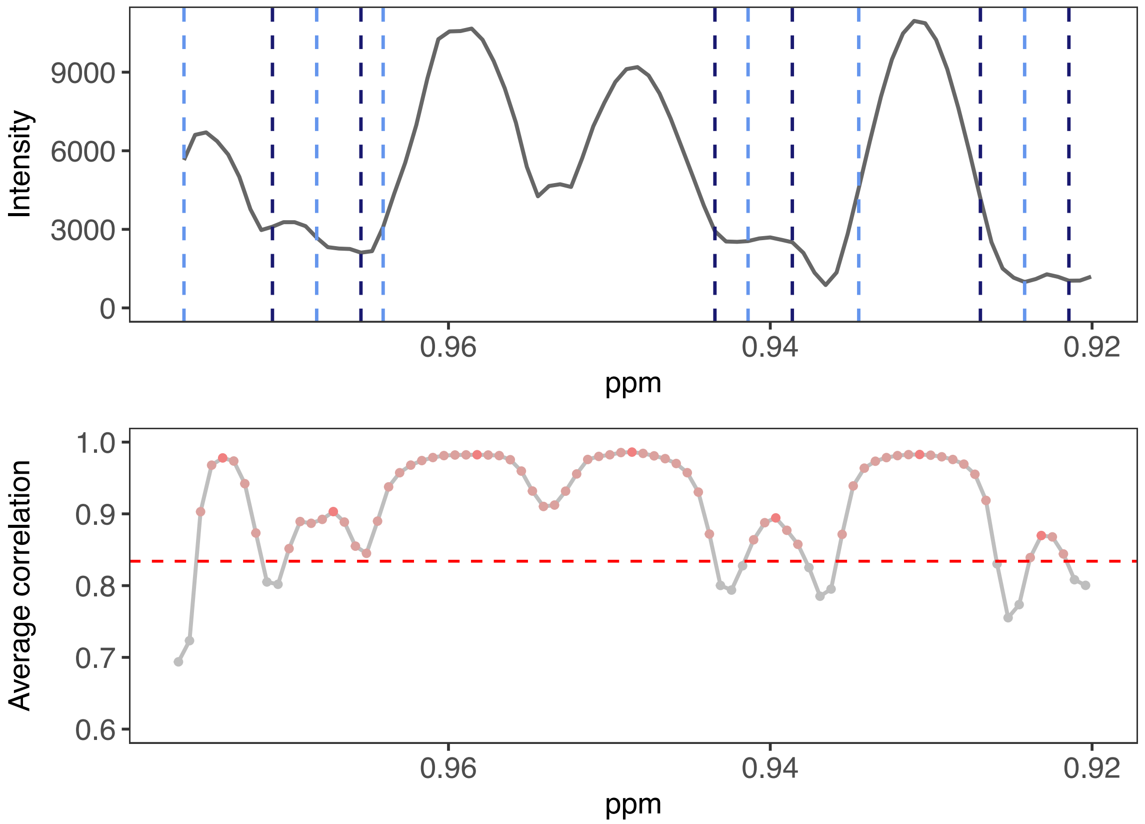

Supplement: bty837_Supplementary_Material [file bty837_supplementary_material.pdf]
